# A microscopic Burgess Shale: small carbonaceous fossils from a deeper water biota and the distribution of Cambrian non-mineralized faunas

**DOI:** 10.1098/rspb.2024.2948

**Published:** 2025-02-19

**Authors:** Giovanni Mussini, Nicholas J. Butterfield

**Affiliations:** ^1^ Department of Earth Sciences, University of Cambridge, Downing Street, Cambridge CB2 3EQ, UK

**Keywords:** Burgess Shale, Cambrian palaeoecology, exceptional preservation, problematica, small carbonaceous fossils, *Wiwaxia*

## Abstract

(SCFs) have disclosed a record of organically preserved faunas from Cambrian epeiric seas. Their phylogenetically and functionally derived components, including probable crown-group crustaceans and molluscs, are absent from the ‘exceptional’ palaeoenvironmental settings captured by Burgess Shale-type (BST) macrofossil biotas. This apparent segregation of SCF and BST-macrofossil deposits has led to contrasting hypotheses on whether their faunal differences reflect genuine ecological patterns or overriding taphonomic controls. We report a new, exceptionally diverse SCF biota from the Cambrian Hess River Formation of the Northwest Territories (Canada), which occupied an offshore slope setting. The Hess River biota, hosted by a single shale sample, rivals the Burgess Shale in its disparity of bilaterian body plans, providing a microfossil counterpoint to the regional record of BST-macrofossil faunas from similar deeper-water palaeoenvironments. The Hess River SCFs comprise exceptionally preserved ecdysozoan and spiralian sclerites, arthropod mouthparts, semi-articulated wiwaxiids, problematica and pterobranchs, but no recognizable crown molluscs or crustaceans. The similarities between the Hess River fauna and classic deeper-water BST-macrofossil biotas suggest significant palaeoecological overlap, robust to their distinct taphonomic expressions. This upholds the existence of comparatively modern communities in Cambrian epeiric settings, distinct from the faunas populating both BST-macrofossil biotas and SCF assemblages sampling similar palaeoenvironments.

## 1. Introduction

Organically preserved, submillimetric small carbonaceous fossils (SCFs; [1]) are increasingly complementing the record of Cambrian faunas provided by their macroscopic counterparts, macroscopic Burgess Shale-type (BST) biotas [1]. As originally defined, BST macrofossils capture non-biomineralized organisms preserved primarily as carbonaceous compressions, often recording whole-body carcasses [2,3]. The exceptional completeness and preservational fidelity of BST macrofossils are typically associated with burial in scarcely bioturbated, oxygen-poor settings sheltered from extensive reworking, decomposition and scavenging [2–4]. SCFs share with BST macrofossils the same underlying mode of carbonaceous preservation: as such, they capture most of the same taxonomic groups, except for those lacking recalcitrant, sclerotized organic body parts [1]. However, the small size and disarticulated state of SCFs confer enhanced biostratinomic potential for transport, burial and resistance to scavenging, expanding the range of Burgess Shale-style preservation well beyond the ‘exceptional’ palaeoenvironments of BST-macrofossil biotas [1,3–5].

**Figure 1.**
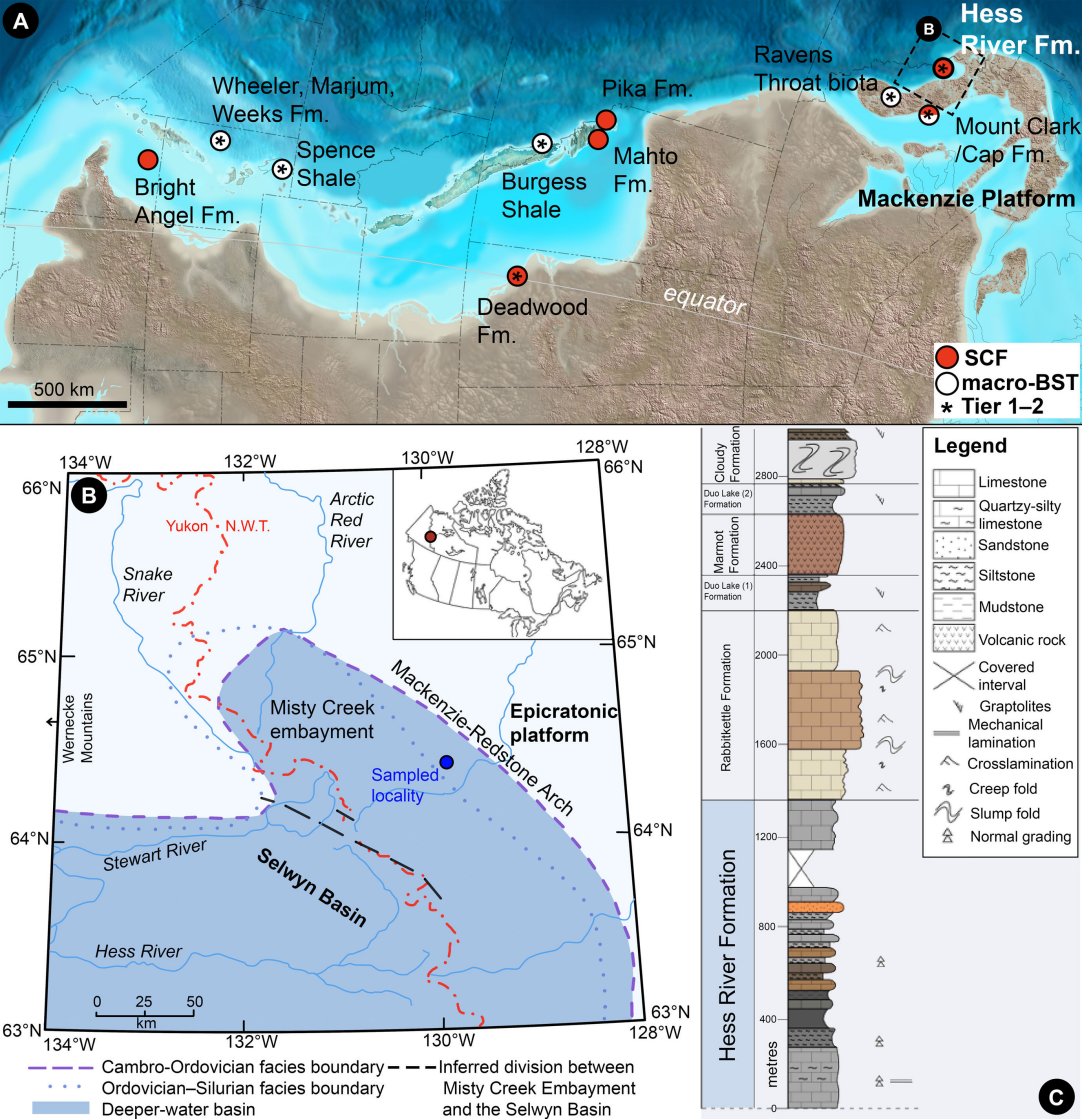
Depositional context of the Hess River (HR) Formation. (*a*) Palaeoenvironmental map of the western edge of mid-Cambrian North America. Dots indicate approximate locations of fossil biotas, after [6–14]. Asterisks denote ‘exceptional’ preservation localities with over 10 preserved taxa and preservation of non-sclerotized body parts (‘Tier 1 to 2’; [3]). Source map © 2023 Colorado Plateau Geosystems. (*b*) Map showing positions of the HR Formation in present-day North America (red circle in the inset) and study area in the Mackenzie Mountains, after [11]. (*c*) Composite stratigraphic section of the Misty Creek Embayment, after [15]. Horizontal scales indicate natural weathering profile. Scaling of pattern fills reflects layering thickness. Colouring fills indicate weathering colour. Fm. Formation; SCF, small carbonaceous fossils.; BST, Burgess Shale-type; N.W.T., Northwest Territories.

The Cambrian of western North America (Laurentia) hosts multiple Cambrian biotas recording ‘exceptional’ preservation of delicate non-biomineralized body structures and over 10 taxa each [3]. Spanning both SCF and BST-macrofossil localities, and distributed along similar palaeolatitudes on the equatorial northern margin of the craton (figure 1A; see also [16–18]), these biotas offer a promising setting to assess the influence of Cambrian palaeoenvironments on faunal composition independently from biogeographic, palaeoclimatic and taphonomic overprints. Even so, the SCF and BST-macrofossil taphonomic ‘windows’ show marked palaeoenvironmental segregation. All known Cambrian SCF biotas from Laurentia record epicratonic or otherwise shallow-water successions: the Earlie [1,19], Deadwood [19–21] and Mahto [6] of the Western Canada Sedimentary Basin (WCSB), the Bright Angel Shale in the Grand Canyon [1], the shelf biota of the Buen Formation of Greenland [16], and the Mount Cap and Mount Clark formations of the Northwest Territories [7,22]. This palaeoenvironmentally biased sampling matches global patterns in the distribution of SCF biotas: the only example of an SCF assemblage from a probable offshore slope, near the lower limits of the photic zone, is the Gondwanan early Cambrian (Stage 4) biota of the Kaili Formation of south China, biogeographically far removed from North American counterparts [23]. In contrast, Laurentian BST-macrofossil biotas largely record distal shelf/slope deposits. The Burgess Shale of the approximately 508 Ma ‘thick’ Stephen Formation of British Columbia captures a deeper water biota facing a steep offshore escarpment, segregated from epicratonic waters to the east by carbonate shoals or patch reefs [24,25]. Meanwhile, assemblages from the ‘thin’ Stephen Formation [26] and the Miaolingian Spence Shale, Marjum, Wheeler and Weeks biotas of Utah and Idaho capture lower-gradient ramps, descending into dysoxic deeper-water basins from neighbouring carbonate platform environments [8,9,27].

Faunal overlap between these BST-macrofossil assemblages and epeiric SCF biotas is correspondingly limited. Like all other known BST-macrofossil biotas, Laurentian examples yield none of the derived crustaceans (branchiopods, copepods and ostracods) and molluscs (putative aplacophorans and/or chitons) typical of the Mount Cap/Clark [7,28], Mahto [6], Bright Angel [1], Earlie and Deadwood [19,20] SCF faunas. Possible crown crustaceans also occur in shelf deposits from the Buen Formation [16]. By comparison, faunas dominated by the stem-group relatives of extant phyla found in BST-macrofossil localities remain under-represented in SCF assemblages [1,10,16]. If these differences reflect genuine biotic signals, Cambrian epicratonic environments emerge as important and largely cryptic ‘cradles’ of later Phanerozoic marine ecologies: well aerated, productive settings that may have fostered the early diversification of extant orders and classes [6,16,20,22], contrasting with the more phylogenetically and functionally archaic faunas of BST-macrofossil biotas. Alternatively, these differences may simply reflect first-order taphonomic controls related to grain resolution limits [29], early diagenetic degradation or greater late-stage thermal alteration [1] hindering the preservation of small, delicate fossils like crustacean molars and setal arrays [29] in BST-macrofossil deposits. If this were the case, SCFs from similar palaeoenvironments to BST-macrofossil biotas—but comparable to ‘epicratonic’ SCFs in their taphonomic expression and level of morphological resolution—may be expected to yield some of the same ‘cryptic’ modern forms.

We test these hypotheses by describing an exceptional offshore, slope-facing Cambrian SCF biota from Laurentia—the Hess River Formation biota. This carbonaceous assemblage, contained within a single exceptionally productive shale specimen, showcases the potential of SCFs to yield rich palaeobiological information even for small quantities of sampled material. Its composition suggests that the differences between BST-macrofossil and SCF biotas track mutually consistent palaeoecological patterns, robust to taphonomic and biogeographical overprints.

## 2. Geological context

The early Palaeozoic passive northwestern margin of Laurentia was occupied by the carbonate-dominated Mackenzie Platform, rimmed by slope-facing troughs and embayments that sustained siliciclastic and deeper-water carbonate deposition [15]. Among them was the Selwyn Basin, extending to the northeast into the Misty Creek Embayment (MCE; [11,15]) of the Northwest Territories of Canada (figure 1B). The MCE was separated from the shallow-water carbonate and siliciclastic environments of the Mackenzie Platform (figure 1A) by a northwest-trending region of tectonic uplift, the Mackenzie-Redstone arch [11,15].

The early/middle Cambrian Hess River (HR) Formation (figure 1C) of the MCE conformably overlies the shelf carbonates and mudstones of the approximately 525–510 Ma Sekwi Formation [11,15] and conformably underlies the lime mudstone-dominated, late Cambrian–Ordovician (approx. 499–471.8 Ma) Rabbitkettle Formation [11,15]. Eastward, it correlates with the glauconitic sandstones, shales and thin-bedded limestone intervals of the Mount Cap Formation from the eastern side of the Mackenzie-Redstone arch [7,11]. The HR Formation encompasses fine-grained carbonates with alternating shale packages, silty limestone and siltstones. Its type section—section 6 of [11]—comprises approximately 60 m of black shale, underlying approximately 360 m of argillaceous limestone and calcareous shale interbeds [11,15]. Alternating recessive and resistant beds, grading, crosslamination and rare groove casts show that the formation records a succession of fine-grained carbonate turbidite units [15]. A lack of thicker turbidite beds suggests deposition on a deeper-water, low-angle slope [15]: a setting similar to that recorded in the successions hosting the Marjum, Wheeler, Weeks [9,30], Kaili [31] and ‘thin Stephen Formation’ [26] biotas. The sedimentological context of the HR formation and its lack of documented bioturbation [11,15] support a calm-water, oxygen-poor environment, comparable to that of these classic BST-macrofossil deposits [3,4,15].

Although largely unfossiliferous except for sparse inarticulate brachiopods, hyoliths, trilobites and spicule beds, the HR Formation is constrained by its assemblage of corynexochid and ptychoparioid trilobites (*Oryctocephalus, Poliella*) to the latest early to middle Cambrian [11]. This age is consistent with carbon isotope stratigraphy evidence for a Wuliuan to mid-Paibian deposition [32].

## 3. Small carbonaceous fossils

The HR biota comprises 968 SCFs (electronic supplementary material, data S1) recovered through hydrofluoric acid maceration from a single field sample of dark grey shale, hand-collected in 1999 at an unknown stratigraphic height in the HR Formation ([11]; electronic supplementary material). Although most of the HR SCFs are essentially two-dimensional compressions, exceptionally robust chitinous sclerites maintain a degree of three- dimensionality. The HR SCFs often associate with pyrite crystals and framboids, suggesting burial in oxygen-poor sediment. Unlike the conspicuously darkened, cracked or otherwise deformed organic microfossils extracted from the Burgess Shale [1], the HR specimens preserve delicate cuticular structures, including setae, scales and cuticular ‘hairs’ down to submicrmetre scales, similar to previously described SCFs from the Mount Clark and Earlie/Deadwood successions [20,28].

**Figure 2.**
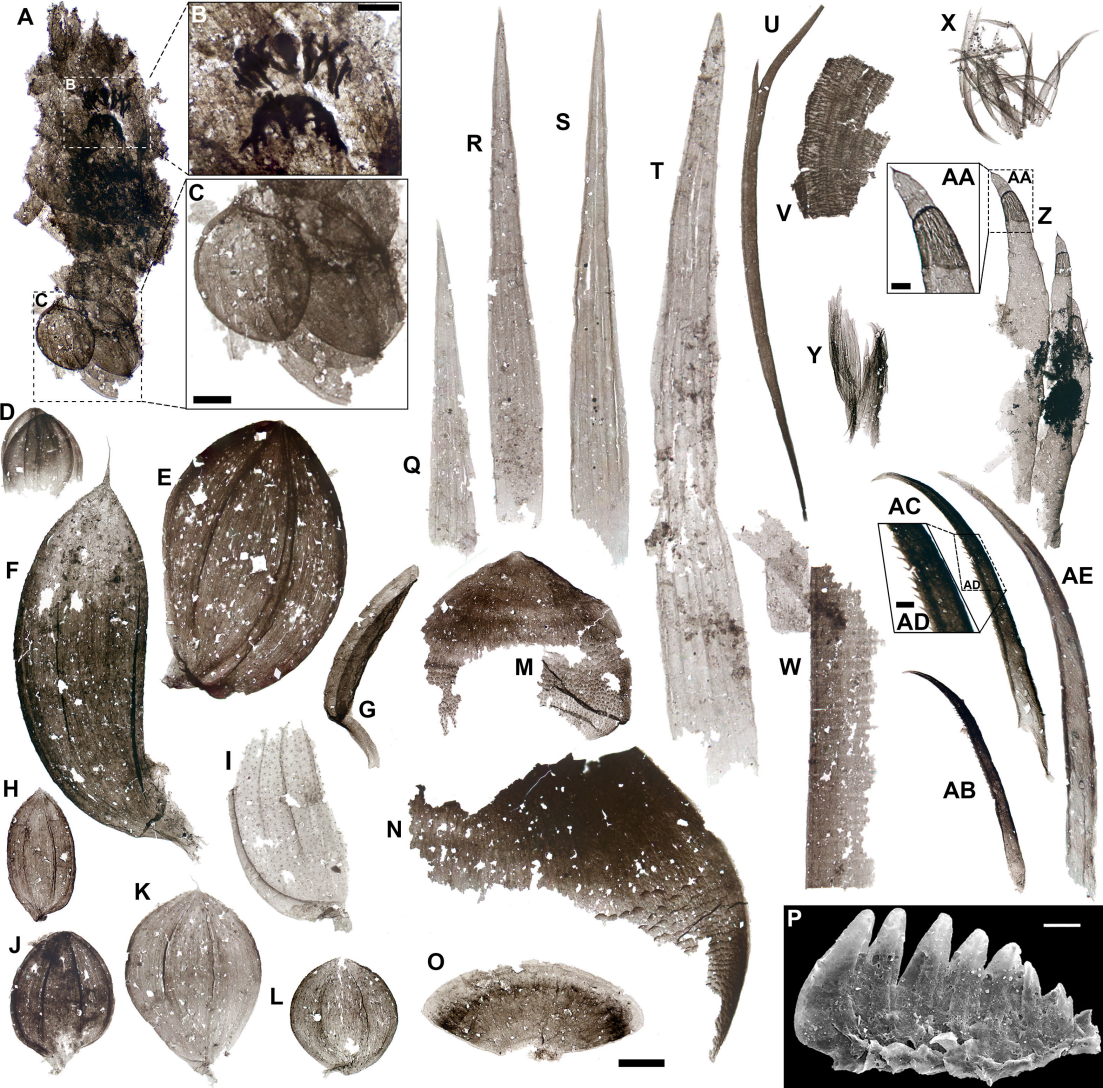
Lophotrochozoans and gnathiferans from the Hess River biota. *A,* Semi-articulated *Wiwaxia*; *B*, detail of biseriate mouthparts in A; *C*, detail of posterior dorsal sclerites in A; *D–L,* probable ventrolateral (*F,G)*, lower lateral (*H*), upper lateral (*D,J,*L) and dorsal (*E,I,K*) *Wiwaxia* sclerites; *M,N*, scaly sclerites from a probable undescribed wiwaxiid; *O*, fan-shaped lophotrochozoan sclerite; *P*, putative gnathiferan jaw (scanning electron microscopy image); *Q–T*, *Wiwaxia* dorsal spines: *U*, bifid annelid chaeta; *V*, brachiopod-type organic shell fragment; *W*, brachiopod-type seta; *X,Y*, chaetognath grasping spines; *Z*, series of chaetognath-like spines with fibrous ‘annuli’; AA, detail of distal portion of spine in Z; AB, AC, grasping spines with serrated inner margins; AD, detail of serrations in AC; AE, non-serrated grasping spine. Scale bars: 50 µm except for B and C (20 µm), P (25 µm), AA and AC (10 µm). Slide numbers and England Finder coordinates are provided in electronic supplementary material, table S1.

**Figure 3.**
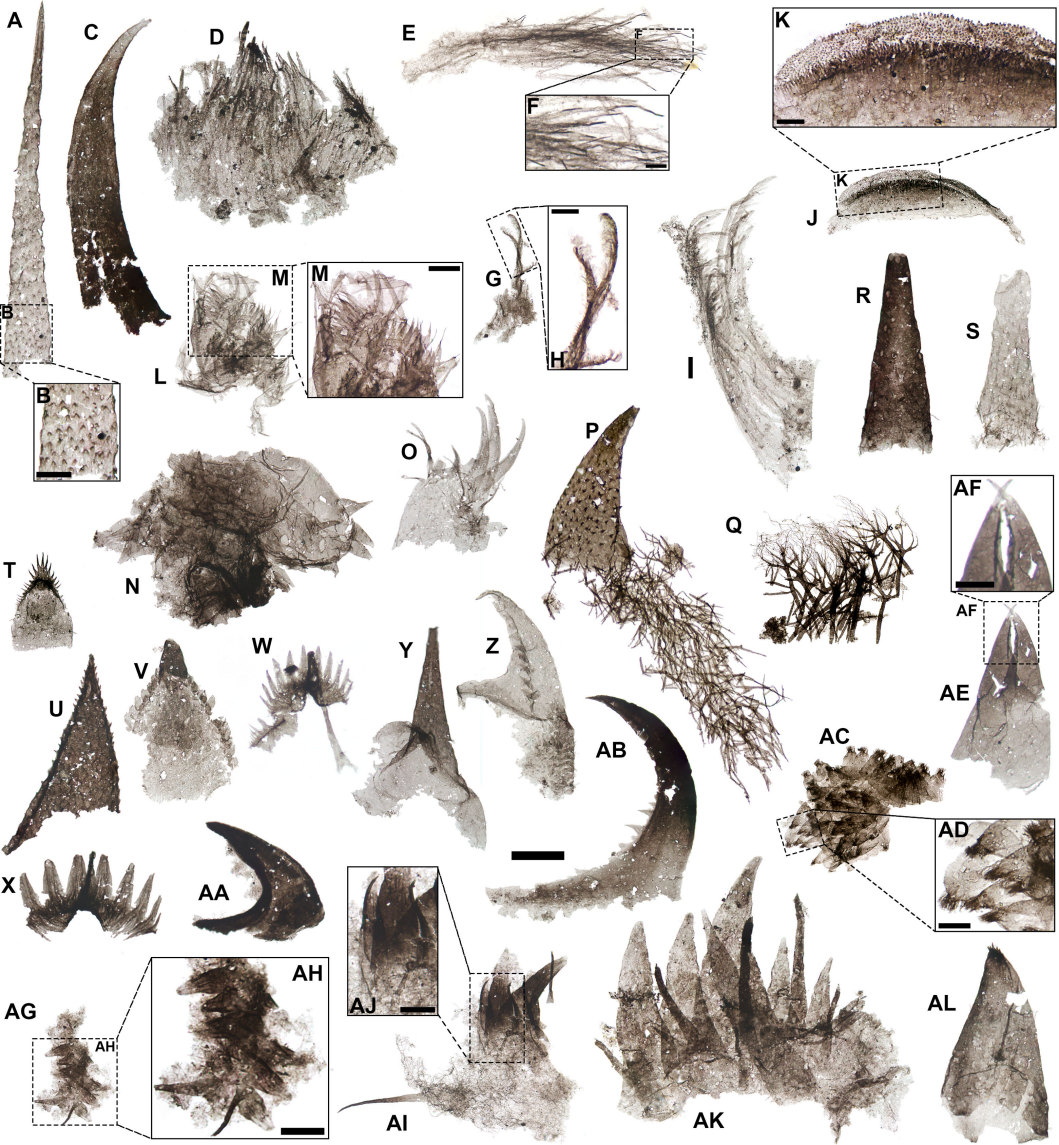
Ecdysozoans from the Hess River biota. *A*, hallucigeniid spine; *B*, detail of A showing spinulose ornament; *C*, possible lobopodian cone—note cone-in-cone construction; *D,E*, setose apparati; *F*, detail of E showing hairs with optically dense tips; *G*, plumose setae; *H*, detail of G showing distal region of plumose setae; *I*, series of apparent recurved setae; *J*, mandibulate-type molar; *K*, detail of J showing scaly ornamentation of molar surface; *I*, series of U-shaped spinule-bearing elements; *M*, detail of L showing distal spinules; *N*, U-shaped elements bearing subconical distal spines; *O*, cuticular base bearing distal claws; *P*, scalidophoran-type sclerite associated with arborescent structures; *Q*, cluster of arborescent structures; *R,S*, coniform sclerites; *T–X*, priapulid-type pharyngeal teeth; *Y*, possible priapulid coronal spine; *Z*, introvert hook with uncinate termination; AA, tail hook; AB, introvert hook with serrate concave margin; AC, possible locomotory scalids; AD, detail of AC showing ‘tufted’ terminations; AE, chelate element; AF, detail of AE showing fang-like terminations and series of darker inner denticles; AG, gnathobase with two series of triangular spines; AH, detail of spines in AG; AI, gnathobase fragment; AJ, detail of spines in AI; AK, large gnathobase lacking fibrous microstructure; AL, subconical apically ‘tufted’ sclerite (cf. AC). Scale bars: 50 µm except for B, M, AG, AI, AK (20 µm), F, H, K, AE (10 µm). Slide numbers and England Finder coordinates provided in electronic supplementary material, table S1.

**Figure 4.**
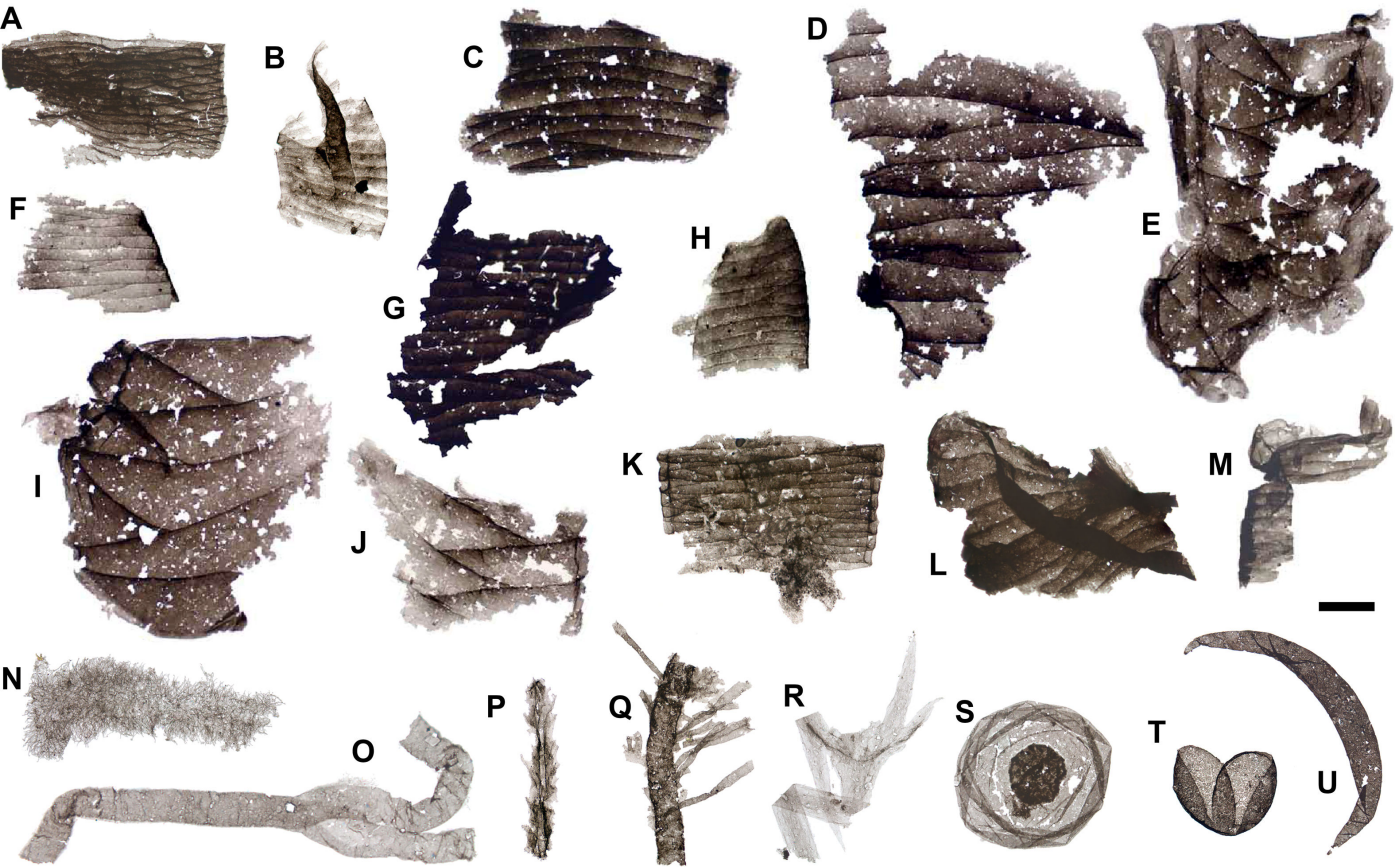
Fragments of pterobranch tubaria and microbial small carbonaceous fossils from the Hess River biota. *A,E,F,I,J,K*, probable graptolithine periderm showing clear zigzag sutures; *B,C,G,H,I,M*, other periderm fragments. *N*, filamentous mat fragment; *O*, bifurcating tubular specimen; *P*, repetitively flaring filament; *Q*, laterally branching strap-like form; *R*, problematic ribbon-like form with calyx-shaped apex; *S,T*, sphaeroidal acritarchs; *U*, lunate acritarch. Scale bars: 50 µm. Slide numbers and England Finder coordinates are provided in electronic supplementary material, table S1.

The HR SCFs comprise 329 recognizably animal microfossils of bilaterian origin, described below (figures 2, 3 and 4A–M). This metazoan record is complemented by 450 carbonaceous microfossils morphologically comparable to bacterial and microbial eukaryotic forms. These diverse SCFs include morphologically exotic branching, calyx-like and nastriform specimens together with sphaeromorphic and lunate acritarchs (figure 4N–U; electronic supplementary material), constituting a significant component of the biodiversity of the HR assemblage and providing evidence for probable benthic and planktonic primary producers. Detailed accounting of these specimens and of problematic metazoan SCFs is provided in the electronic supplementary material.

### (a) Wiwaxiids

The most common metazoan SCFs in the HR biota are sclerites produced by wiwaxiids, a group of slug-like, sclerite-bearing Cambrian and potentially Ordovician lophotrochozoans [33–36]. Like those of most other lophotrochozoans—the clade comprising annelids, molluscs, brachiopods and their relatives—*Wiwaxia* sclerites show a diagnostic internal microstructure of fine parallel fibres, denoting microvillar secretion [1]. Wiwaxiid body sclerites have a diagnostic leaf-like shape with a hollow stem and a flat, ovate distal surface with longitudinal ribs [33]. The HR specimens (*n* = 57) are approximately 90–480 µm long and include asymmetrical dorsal sclerites (figure 2E,I,K; electronic supplementary material, figure S1 Y–AB); rounded, symmetrical upper-lateral sclerites (figure 2D,J,L; electronic supplementary material, figure S1 U–X); ovoid, symmetrical lower lateral sclerites (figure 2H; electronic supplementary material, figure S1 L–T) and sickle-shaped ventrolateral sclerites (figure 2F-G; electronic supplementary material, figure S1 A–K) [33,35]. The number of longitudinal ribs on each sclerite varies from 2 (e.g. figure 2H,J–L) to 11 (e.g. electronic supplementary material, figure S1 R), overlapping with the *n* = 2–9 range of *Wiwaxia corrugata* juveniles [35]. A pustulose ornamentation, typical of *W. corrugata* juveniles shorter than 8 mm [35], is occasionally visible (figure 2H,I,K,L; electronic supplementary material, figure S1 K,M,R,S,W). The HR assemblage also preserves *Wiwaxia* dorsal spines (*n* = 39): approximately 195–850 µm long, slender, basally incomplete acuminate sclerites marked by thickened margins, a fibrous construction and 3−5 parallel longitudinal ribs (figure 2Q–T), directly comparable in shape and ornamentation to macrofossil counterparts (fig. 1D in [33,35]). The absence of dorsal spines in *W. corrugata* juveniles, their higher minimum number (*n* = 5) of longitudinal ribs [35] than in the HR fossils, and the occurrence of juvenile characters such as pustules and low rib counts in the HR sclerites suggest a distinct species-level attribution, with spines appearing earlier during ontogeny in the HR *Wiwaxia* than in *W. corrugata*.

The Hess River SCF assemblage includes a unique semi-articulated slab of *Wiwaxia* sclerites and associated mouthparts. This 450 µm long fragmentary scleritome (figure 2A) falls below the >2 mm size range of known wiwaxiid macrofossils [33,35] and records approximately 15−20 individual plates. Anteriorly, mouthparts with two U-shaped tooth rows are visible. The tooth rows inflect towards the anterior end of the animal (figure 2B). The anterior-most row consists of a broader, subconical pointed tooth medially, flanked on each side by six peg-like teeth (cf. [34], figure 4A). The second row is more markedly arcuate than the first and shows similarly peg-like but somewhat broader, more densely spaced, basally abutting teeth (figure 2B).

Other, incomplete chaetae share with *Wiwaxia* sclerites a fibrous construction and broad leaf-like outlines, but fall outside the known range of variability of the genus [33,35,37]. The surface of these sclerites (*n* = 10) shows acuminate, distally pointing, <15 µm long equilateral scales basally, distributed into irregular transverse rows (figure 2M,N; electronic supplementary material, figure S1 AC–AG). This ornamentation fades abruptly about midway along the length of the chaeta, leaving a ‘naked’ conspicuously fibrous apical portion with a broad isosceles tip (figure 2M). The large surface pustules observed in *Wiwaxia* SCFs from the Pika Formation of Alberta [12, fig. 11F,G] provide partial morphological counterparts despite their lower density and more elongate shapes, suggesting that the ‘scaly’ HR sclerites belong to an unknown wiwaxiid. A scleritome-bearing lophotrochozoan is also the likely producer of a rigid, fan-shaped chaeta with a fibrous microstructure and a distinctive ‘banded’ coloration, lighter distally (figure 2O).

### (b) Annelids

Three chaetae from the HR assemblage are attributable to annelids based on morphological correspondences with modern polychaete counterparts. Two elongate, 410−600 µm long strap-like chaetae show bifid tips with two pointed, asymmetrical prongs, one up to three times the length of the other (figure 2U; electronic supplementary material, figure S3 F). These specimens resemble the ‘lyrate’ [38] chaetae of several extant annelid families (e.g. [39, fig. 16B;^40^, fig. 4A–C;^41^, fig. 3]). In addition, they are similar in size and morphology to macrofossil and SCF counterparts from the Cambrian annelid *Burgessochaeta* [12,42].

**Figure 5.**
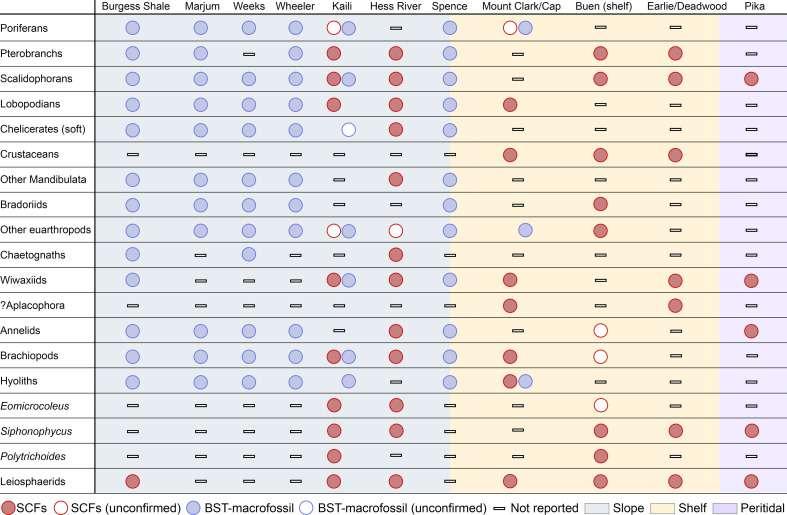
Palaeoenvironmental settings and representative taxonomic occurrences for the main Laurentian small carbonaceous fossil (SCF) and Burgess Shale-type (BST) macrofossil biotas, together with the Gondwanan Kaili Formation, which hosts a probable deeper-water SCF biota comparable to that of the Hess River Formation. Colour-coded legend (bottom) indicates taphonomic and environmental occurrences of each taxon. Data from [1,2,6–9,12,13,16,18–20,23,25,27,30,46,80,81,84,86–92].

A stiff, weakly recurved and basally and apically incomplete chaeta approximately 760 μm long and 120 wide (electronic supplementary material, figure S3 G) shows a marked fibrous construction and a series of widely spaced, distally directed triangular spines on its convex edge. This ornamentation matches that of ‘serrate’ chaetae in extant polychaete families as diverse as nephtyids [43, figs 2–6], pilargids [44, fig. 3] and nereidids [45, fig. 3F], supporting an annelid producer.

### (c) Brachiopods

Lophotrochozoan SCFs from the HR biota include rare brachiopod elements. A thin-walled, 185 µm wide fragmentary sheet shows a concentrically banded fabric and a transverse pattern of fibrous lineations (figure 2V). This SCF most likely records the periostracum or internal organic layers of brachiopod shells, which show the same style of concentric ornament [23,46]. Another six 225−565 µm long SCFs of probable brachiopod origin consist of strap-like setae with regularly spaced (approx. 12–75 µm apart) faint transverse bands, yielding an overall bamboo-like appearance (figure 2W; electronic supplementary material, figure S3 A−E). These bands are not associated with lateral notches/indentations, unlike the more pronounced transverse stripes characterizing the multi-articulated chaetae of cirratuliform annelids [12]. Closer matches to the HR specimens are the setae of lingulid brachiopods, which show similar continuous straight-sided edges and subdued transverse patterning (fig. 5I in [47]).

### (d) Chaetognaths

Chaetognaths are pelagic arrow-shaped worms bearing chitinous ‘grasping spines’, normally retracted into an oral hood and projected forward for prey capture. Grasping spines have a distinctive three-layered microstructure comprising a laminated inner layer, a fibrillar middle layer and a thin, possibly laminated outer layer [48]. Twenty-three SCFs of grasping spines from the HR biota are readily recognizable by their sickle-shaped profile, acuminate apices, laterally compressed ‘blade-like’ configuration and concave margin expressed as a sharp keel (figure 2X,Y,AB–AE; electronic supplementary material, figure S3 I–M). The HR grasping spines occasionally are preserved in semi-articulated bundles denoting an original bilaterally symmetric arrangement (figure 2Y), with two opposite spinose arrays (cf. [49, fig. 10]) converging anteriorly towards the oral midline (cf. [50;51, fig. 4]).

The keels of the HR grasping spines are either smooth (figure 2AE; electronic supplementary material, figure S3 I–M) or serrated (figure 2X, AB–AD). Serrated spines bear a series of finely spaced, disto-laterally pointing denticles on their basal (figure 2X) or median portions (figure 2AB,AC). Smooth keels characterize most extant chaetognath genera [48,52], the Burgess Shale taxon *Capinatator* [50], previously described Cambrian chaetognath SCFs [53,54] and ‘small shelly fossils’ (e.g. [51]). In contrast, inner marginal denticles occur in the extant *Serratosagitta* and *Eukrohnia* [55–57]. Serrated spines also typify the Carboniferous chaetognath *Eoserratosagitta* [55]. The HR fossils represent the earliest known record of the serrated habit, showing that Cambrian chaetognath spines had attained a range of basic architectures comparable to that of later Phanerozoic counterparts.

Another putative chaetognath SCF records a series of three 280−375 µm long spines (figure 2Z). Up to 56 µm wide, these weakly curved elements each bear a short apical spinule and a subterminal annular band. The ‘band’ consists of a region of robust, tightly spaced and optically dense longitudinal fibres. The bundled arrangement and arcuate shape of the sclerites in this specimen are reminiscent of chaetognath grasping spines, and finely tapering, spindle-like terminations are seen in the recent taxon *Sagitta* (figure 2AA cf. [48, fig. 6A1,A2]). In extant chaetognath spines, a ‘fibrous’ appearance stems from a localized absence of the outer lamellar cover, exposing the underlying fibrillar construction of the ‘middle’ layer; a similar fibrous architecture has also been identified in the Cambrian ‘protoconodont’ chaetognath *Phakelodus* [48, fig. 9]. Even so, the HR specimens differ in having the exposed fibrous portion subapically rather than terminally [48], potentially suggesting different forms of dietary wear from those in extant counterparts.

### (e) Other gnathiferans

Chaetognath spines and *Wiwaxia*’s biseriate feeding apparatus contrast with an isolated, 210 µm long series (HR-0089B) of 7 or 8 robust, lunate to subtriangular teeth with blunt apices (figure 2P). These teeth are approximately 18–90 µm long, entirely opaque and conspicuously three-dimensional. They are unfused laterally but welded at the base, yielding a single ‘denticulate plate’ (cf. [34]) with ragged, potentially broken margins basally. The teeth decrease in size unilaterally. Their lateral curvature is strongest in the largest element and decreases as tooth size declines (figure 2P).

The basally fused and abutting uniseriate teeth, unilateral gradient in tooth size and tapering profile of HR-0089B are morphologically reminiscent of the jaws of gnathostomulids—a phylum of acoelomate meiofaunal worms armed with cuticular mouthparts. However, gnathostomulid teeth do not arch laterally, but rather perpendicularly to the jaw’s longitudinal axis [58, fig. 8F]. Like those of the closely related, similarly miniaturized micrognathozoans, gnathosomulid jaws are also an order of magnitude smaller than HR-0089B [59]. A closer match is the mouthparts of the Burgess Shale gnathiferan *Amiskwia* [60]. *Amiskwia* jaws comprise two robust bilaterally symmetrical plates, each with a continuous base supporting a series of recurved teeth on the anterior margin. The teeth arch and typically decrease in size towards the animal’s midline (fig. 5C,F in [60]. The absence in HR-0089B of the postero-laterally projecting ‘rod’ observed in *Amiskwia* jaws [60, fig. 5D] is compatible with its ragged basal edge, potentially denoting taphonomic breakage of all but the most sclerotized toothed parts of this oral apparatus (figure 2P). *Amiskwia*’s approximately 400 µm long jaws also fall within the same general size range as the approximately 210 µm long HR-0089B. These similarities suggest that HR-0089B was produced by an *Amiskwia*-like gnathiferan.

### (f) Scalidophorans

Scalidophorans are a group of benthic, radially symmetric, anatomically tripartite ecdysozoans comprising priapulids, loriciferans and kinorhynchs [61]. Their sclerites have a characteristic internally hollow structure and typically show denticular or spinose patterns of surface ornamentation [62,63].

The HR scalidophorans include nine 95−215 µm long sclerites with diagnostic characters of the ‘teeth’ lining the priapulid pharynx: triangular to U-shaped profiles, a basal cuticular pad, a marginal arch with distally directed denticles and occasionally a marked distal prong and/or a basal spur [13,63]. The denticles on these sclerites range from fine serrations on semi-triangular elements (figure 3U) to ovoid spinulose pinnules (figure 3V), long subapical spines (figure 3T) and spear-shaped counterparts borne on a gently curved arch (figure 3W,X); the latter are reminiscent of ‘Type B’ teeth in the Burgess Shale priapulid *Ottoia* [13,63]. Smaller (approx. 35 µm long) elements with apical clusters of spinose to filiform denticles, lacking a diagnostic prong, sclerotized arch or basal spur [13], may represent ‘trunk scalids’ similar to those of *Meiopriapulus* (figure 3AC–AD, cf. fig. 3C in [64]).

Falciform sclerites with acute denticles on their concave margin, a basal spur and occasionally uncinate terminations (figure 3Z,AB) can be identified as priapulid introvert ‘hooks’ [13,63]. Similarly robust, sickle-shaped sclerites with a marked spur, but showing an excavate concave surface and lacking denticles (figure 3AA), likely represent ‘tail hooks’ from the posterior end of the priapulid body [13]. In contrast, straight-sided sclerites tapering to a fine point distally and showing a subtriangular basal opening (figure 3Y) resemble the ‘coronal spines’ fringing the introvert in *Ottoia* macrofossils [13, fig. 4F].

Other scalidophoran SCFs from the HR assemblage comprise hollow, hook-like to coniform sclerites with dense spinules on their outer surface (figure 3P,S), recurrently associated with arborescent cuticular structures (cf. figure 3Q). These SCFs, attributed to the taxon *Scalidodendron crypticum*, are described in a separate contribution [65].

### (g) Panarthropods

Panarthropods, including onychophorans, tardigrades and euarthropods [61], constitute the most diverse category of SCFs in the HR biota after lophotrochozoans (electronic supplementary material, data S1).

Lobopodian stem-group onychophorans [66] or panarthropods [67] are recorded by hallucigeniid spines (*n* = 6). These fossils are identified by a layered ‘cone-in-cone’ onychophoran-type construction [66], straight-sided and distally tapering shapes and dense distally directed surface spinules (figure 3A, B). The comparable cone-in-cone structure of a co-occurring hook-like, unornamented approximately 330 µm long sclerite (figure 3C) suggests that it records the claw of a similar lobopodian-grade producer [66].

Euarthropods (*n *= 10) are represented by more diverse sclerites. The most extensively articulated consist of three series of triangular to arcuate spines of variable length. In these specimens, the spines line an optically light base of loose fibrous tissue and are flanked by sparse, elongate spinules (figure 3AG,AI,AK). In two specimens (morphotype 1), the spines bear fibrous longitudinal striations and are optically densest basally, becoming progressively lighter apically (figure 3AH,AI). In the smallest morphotype 1 specimen, 115 µm long, the spines form two parallel but bilaterally asymmetrical series, splayed laterally (figure 3AG,AH). A larger (approx. 290 µm long) specimen (morphotype 2; figure 3AK) shows thinner-walled and more optically homogeneous spines, lacking visible fibrous microstructure and borne on an arcuate base. By comparison with macrofossil [68, fig. 3;^69^, figs 6,8,9;^70^, fig. 6A] and extant [69, figs 2,4,11] counterparts, morphotypes 1 and 2 are identified by their lateral asymmetries, dentate margin, serialized acuminate spines or ‘teeth’ and flanking accessory spinules as gnathobases—the proximal masticatory and manipulatory processes of arthropod appendages. Although these structures occur widely among Cambrian arthropods (e.g. [71]), the gnathobases most similar to the HR specimens, combining a toothed arcuate edge with slender flanking spinules, occur in chelicerates. Counterparts occur in eurypterids [70, fig. 6A], horseshoe crabs [72, fig. 9D] and the potential Cambrian vicissicaudate *Sidneyia* [69]. Tooth microstructures of longitudinal fibres denote durophagy in the gnathobases of Cambrian to Recent chelicerates [69]. Their absence in the least optically dense and sclerotized of the HR gnathobases—morphotype 2 (figure 3AK)—suggests a corresponding diet of softer preys [69].

Chelicerate producers for the HR gnathobases are consistent with the co-occurrence of a chelate (pincer-like) SCF (figure 3AE). This approximately 185 µm long and up to 96 µm wide fossil shows a broad trapezoidal base accommodating two recurved distal claws, converging distally and facing each other as mirror-like opposites. One claw is a direct extension of the base; the other is accommodated into it by a socket. Each claw shows a denticulate inner margin and a lighter, basally indented fang-like termination (figure 3AF). The chelate morphology, optically lighter ‘fangs’ and the potentially denticulate margin of the HR specimen find counterparts in the similarly sized (approx. 50 µm long) chelicerae of *Mollisonia* from the Burgess Shale [73, fig. 2a,b, extended data fig. 3a].

Other HR arthropod mouthparts denote mandibulate producers. The finely denticulate ‘scaly’ surface, crescentic outline and pronounced curvature of one approximately 190 µm long SCF (figure 3J,K) identify it as a molar surface—the apical grinding portion of a mandibular complex [28,74]. Molars with fine, scaly surfaces occur in extant crustaceans, myriapods and basal hexapods [74]. Among Cambrian macrofossils, specimens of the hymenocarine *Branchiocaris* from the Marjum Formation show mandibular appendages with similar curved profiles and finely lineated scaly surfaces, and otherwise lacking any distinctive patterns of ornamentation [75, fig. 2k, extended data fig. 8]. The same morphology may also characterize the closely related *Tokummia*, from the Burgess Shale [75, fig. 2d–f]. A hymenocarine producer for the HR molar is suggested by the lack of any diagnostic features seen in unambiguous crustacean counterparts, including marginal setose fringes, lateral gradients in scale size and ornamentation, marginal teeth and/or oblique spinulose fields [16,20,28]. Hymenocarine origins are also consistent with the lack of any associated sternal groove sclerites and filter plates. These elements are unknown in hymenocarines but found in branchiopods, copepods, malacostracans and amphipods, and invariably preserved in known SCF biotas yielding crustacean molars [16,20,28].

Probable mandibulate SCFs from the HR biota also comprise arcuate pad-like elements bearing uniseriate subconical spines (figure 3L–N) or falciform claws and flanking spinules (figure 3O) distally. In one specimen, these sclerites articulate into a multi-element complex with distal spines tapering into elongate filaments (figure 3L,M): this morphology is comparable to hymenocarine mandibular palps [76, fig. 8G,H;^77^, figs. 9f, 21]. The element bearing falciform spines (figure 3O) also finds a potential match in hymenocarines: similar ‘clawed’ and spinulose arrays occur on the rounded terminations of post-maxillular appendages in *Waptia* [77, fig. 11g–i]. A *Waptia*-type producer is also suggested by a large (approx. 800 µm long), lanceolate SCF fringed by marginal setules (electronic supplementary material, figure S4 K,L), similar in shape and size to the post-cephalothoracic lamellate appendages of the genus [77].

Other SCFs of putative arthropod provenance are clusters of setae, ranging from simple hairs with optically dense tips (figure 3D–F) to plumose counterparts (figure 3G,H) and elongate structures borne on an axial support (figure 3I, cf. [77, fig. 7]). Since these setal bundles lack the distinctive coplanar arrangement, mesh-like substructure and arcuate outlines of crustacean filter plates [16,20,28], they may belong to non-crustacean hymenocarines [77] or even non-mandibulates [e.g. [78]].

### (h) Hemichordates

Pterobranchs (the hemichordate class comprising Cephalodiscida and Graptolithina) share a diagnostic collagenous periderm or ‘tubarium’. The tubarium is defined by a fusellar construction with banded, oblique to truncated sutures [30,79]. In graptolithines, the fuselli indent each other to give the sutures a characteristic zigzag profile [79]. Fusellar microstructures are visible in 95≤285 µm wide organic fragments from the HR biota, identifying them as parts of pterobranch periderm. The co-occurrence of specimens with simple truncated (e.g. figure 4B,C,G,L,M) and indented, zigzag sutures (e.g. figure 4E,I,J) suggests the presence of—minimally—two taxonomically distinct forms, a graptolithine and a non-graptolithine.

## 4. Discussion

The HR assemblage records the first known Laurentian SCF biota facing a deep-water basin. As such, it provides a microfossil counterpoint to the Burgess Shale and other classic deeper-water Konservat-Lagerstätten from the palaeocontinent’s western margin (figure 1A), bypassing the usual palaeoenvironmental segregation of SCF and BST-macrofossil biotas. Its depositional context—a slope descending into oxygen-poor, oceanward basinal environments—parallels that of the Marjum, Wheeler, Weeks [9,30] and ‘thin Stephen Formation’ [26] assemblages. Uniquely, this combined palaeoenvironmental and taphonomic expression of the HR biota permits a comparison of Cambrian faunas under the SCF and BST-macrofossil ‘windows’ [1] from similar depositional settings and palaeolatitudes, testing the impacts of palaeoecological variation on their compositional differences.

The disparity of the HR fauna—captured by a single exceptionally rich shale sample from one locality (figure 1C)—matches or exceeds that of the most taxonomically varied SCF assemblages so far reported. By comparison, the Mount Cap/Clark succession of northern Canada’s Mackenzie Platform has yielded SCFs from approximately five animal phyla: arthropods, brachiopods, molluscs, onychophoran-grade hallucigeniids and chancelloriids—as well as the problematic hyoliths [22,28,80,81]. The similarly diverse Earlie/Deadwood succession, sampled across at least 13 middle to late Cambrian localities of the WCSB, has yielded SCFs from six phyla: arthropods, priapulids, loriciferans, molluscs, pterobranchs and chordates (i.e. paraconodonts; [19–21]). The HR biota records all these groups except chordates and loriciferans, together with annelids, brachiopods, onychophoran-grade lobopodians and chaetognaths (electronic supplementary material, data S1). Of 12 bilaterian phyla reported in the Burgess Shale—the most diverse Laurentian BST assemblage [3]—only the non-sclerotized echinoderms, enteropneusts and chordates ([25,82,83] cf. [84]) remain unrecorded in the HR biota.

Against the background of this relatively comprehensive metazoan record, the HR biota emerges as compositionally distinct from Cambrian epicratonic SCF assemblages. In particular, it yields none of the derived, functionally ‘modern’ crustaceomorphs commonly reported from the Earlie/Deadwood succession [19,20], the Mount Clark Formation [22,28,80] and the Buen Formation of Greenland [16], among which are probable crown crustaceans [20,28,80]. The lack of crustacean SCFs is not readily explainable by the proposed taphonomic ‘filters’ of thermal alteration and/or grain size, which vary considerably within the macro-BST taphonomic window [1,29]. The submicrmetre-scale resolution of delicate setae (figure 3H), molar scales (figure 3K), hairs (electronic supplementary material, figure S4 L) and other fine cuticular specializations (figure 3P,Q,AD) in the HR specimens is comparable to that of ‘exceptional’ epicratonic SCF biotas [1], which preserve similar fine-scale structures in recognizable crown-group crustaceans [1,16,20,28,80]. Similar to SCFs from the Mount Clark, Earlie/Deadwood and Buen biotas, the HR specimens also lack the severe thermal alteration and/or deformation observed in carbonaceous microfossils from the Burgess Shale [1], the Mahto Formation [6] or—to a lesser extent—the Kaili Formation [23]. Despite this overlap in taphonomic expression and scale of morphological resolution with classic ‘shallow-water’ SCF biotas, none of the HR specimens records diagnostic crustacean morphologies like filter plates, lateral setal fringes, sternal grooves or asymmetrically patterned molars [16,20,28,74,80].

With the caveat that the HR biota is presently known from a single sample, the differences between its assemblage and shallow-water counterparts challenge overriding taphonomic explanations for the distinct composition of SCF and BST-macrofossil faunas. Coarser grain sizes and/or more pronounced thermal alteration in BST-macrofossil deposits, which lack recognizable crustaceans [1,29], would not explain why these arthropods are also absent among the comparatively unaltered HR SCFs—but do occur in epicratonic biotas expressing an equivalent mode of preservation. At the same time, taphonomic hypotheses fail to explain why whole-body carcasses from non-crustacean mandibulates and other arthropods are readily preserved in BST-macrofossil deposits, but those of similarly centimetre-sized and sclerotized crown crustaceans like the producers of the Mount Clark and Deadwood molars [20,80] do not. Likewise, overriding taphonomic explanations for the differences between SCF and BST-macrofossil biotas do not account for the Cambrian distribution of functionally modern, multi-echelon molluscan radulae [1,6,7,19,85]. Like derived crustaceomorphs, these elements are recurrent in epicratonic SCF faunas [1,6,7,19,85], not least in the thermally altered Mahto assemblage [6]. However, no such radulae were found in the HR biota. The greater robustness and three-dimensionality of these tooth-bearing elements compared with crustacean scales or setae [1]—and the demonstrable preservation of similar chitinous lophotrochozoan mouthparts in both the Burgess Shale [34] and the HR biota (figure 2B)—argue against a first-order taphonomic explanation for their absence in BST-macrofossil deposits. Furthermore, the potential for both macro-BST and SCF carcasses to undergo significant transport [1,5] does not explain why less phylogenetically or functionally modern forms should be systematically displaced to deeper-water facies, regardless of carcass size.

The distinctive faunal signature of the HR biota, supported by the absence of derived forms typical of other SCF localities, is strengthened by its marked taxonomic overlap with deeper- water macrofossil Konservat-Lagerstätten (figure 5). Scalidophoran worms, wiwaxiids, brachiopods and lobopodians coexisted with derived crustaceans and molluscs in some Cambrian epeiric faunas [1,7,19,81]. However, among SCF assemblages, their mutual co-occurrence and association with pelagic gnathiferans and non-crustacean, poorly sclerotized macroarthropods are unique to the HR biota (figure 5). In contrast, all these groups are well represented in BST-macrofossil faunas from deeper-water, dysoxic environments [3,9,25,82]. Non-biomineralized chelicerate and hymenocarine-grade arthropods from the HR biota find direct counterparts in the Burgess Shale [73,75,77], Wheeler [93] and Marjum [94,27] biotas but are altogether unknown (figure 5) in previously described Cambrian SCF faunas [1,19,20,22,95]. Tube-dwelling pterobranchs, making up approximately 10% of recovered SCFs in the HR biota (electronic supplementary material, data S1), are rarer in the Burgess Shale, where they constitute <0.1% of the slope-facing Great Phyllopod Bed community [30,82]. In keeping with the recovery of tubaria in the Buen [18] and Deadwood [19] biotas, this might suggest that Cambrian pterobranchs were more prevalent in shallow-marine settings [30]. Nonetheless, pterobranchs are well represented in the deeper-water Marjum biota, where 55 out of approximately 200 reported non-biomineralized fossils record their tubaria [30].

Similarly, pterobranchs co-occur with typical Burgess Shale taxa like wiwaxiids, priapulids and lobopodians in the SCF biota of the Kaili Formation [23]. Despite its palaeogeographical remoteness, lower diversity of body plans and somewhat higher degree of thermal alteration [23], this Gondwanan SCF assemblage shows greater taxonomic overlap with the HR biota than do epicratonic faunas from the Mackenzie Platform or the WCSB (figures 1A and 5). Like the HR assemblage, the Kaili SCF biota lacks recognizable crown molluscs or crustaceans, and its fauna is dominated by ecdysozoan worms, bilaterian problematica and scleritome-bearing lophotrochozoans [23]. The overlap between the HR and Kaili biota is not restricted to metazoans. Instead, it extends to probable photosynthetic bacteria and algal forms (figure 5; electronic supplementary material), consistent with a similar depositional and palaeoecological setting near the lower limits of the photic zone [23,31]. These correspondences between two SCF biotas far apart in space, but occupying similar deeper-water settings, support an overriding palaeoenvironmental influence on their taxonomic makeup.

Overall, the composition of the HR biota suggests that discrepancies between classic BST Konservat-Lagerstätten and SCF faunas from different depositional settings capture genuine, mutually consistent biotic patterns. This points to first-order ecological controls on the distribution of Cambrian organically preserved faunas, upholding SCFs and their macroscopic counterparts as overlapping, complementary windows on early Phanerozoic biotas. A distinction between Cambrian epeiric ‘cradles’ of extant orders and classes, and more phylogenetically ‘archaic’ Burgess Shale-like biotas from deeper-water, dysoxic or otherwise marginal settings [12] is supported as a palaeoecological phenomenon robust to taphonomic or biogeographical overprints. In keeping with proposed Phanerozoic-wide trends of nearshore clade origination followed by offshore displacement, the drivers of this pattern may ultimately be sought in differential dispersal patterns or in the links between rates of adaptive innovation and the enhanced habitat heterogeneity, productivity and metabolically permissive conditions of marine shelf settings [96,97].

## Data Availability

All data are presented in the manuscript and the online electronic supplementary material [98].
